# Size Constancy Mechanisms: Empirical Evidence from Touch

**DOI:** 10.3390/vision6030040

**Published:** 2022-07-01

**Authors:** Luigi Tamè, Suzuki Limbu, Rebecca Harlow, Mita Parikh, Matthew R. Longo

**Affiliations:** 1 School of Psychology, University of Kent, Canterbury CT2 7NP, UK; 2 Department of Psychological Sciences, Birkbeck, University of London, London WC1E 7HX, UK; suzukilimbu@gmail.com (S.L.); rebecca_harlow@hotmail.com (R.H.); mita_1994@live.co.uk (M.P.)

**Keywords:** touch, anisotropy, body representations, size, distance, constancy

## Abstract

Several studies have shown the presence of large anisotropies for tactile distance perception across several parts of the body. The tactile distance between two touches on the dorsum of the hand is perceived as larger when they are oriented mediolaterally (across the hand) than proximodistally (along the hand). This effect can be partially explained by the characteristics of primary somatosensory cortex representations. However, this phenomenon is significantly attenuated relative to differences in acuity and cortical magnification, suggesting a process of tactile size constancy. It is unknown whether the same kind of compensation also takes place when estimating the size of a continuous object. Here, we investigate whether the tactile anisotropy that typically emerges when participants have to estimate the distance between two touches is also present when a continuous object touches the skin and participants have to estimate its size. In separate blocks, participants judged which of two tactile distances or objects on the dorsum of their hand felt larger. One stimulation (first or second) was aligned with the proximodistal axis (along the hand) and the other with the mediolateral axis (across the hand). Results showed a clear anisotropy for distances between two distinct points, with across distances consistently perceived as larger than along distances, as in previous studies. Critically, however, this bias was significantly reduced or absent for judgments of the length of continuous objects. These results suggest that a tactile size constancy process is more effective when the tactile size of an object has to be approximated compared to when the distance between two touches has to be determined. The possible mechanism subserving these results is described and discussed. We suggest that a lateral inhibition mechanism, when an object touches the skin, provides information through the distribution of the inhibitory subfields of the RF about the shape of the tactile RF itself. Such a process allows an effective tactile size compensatory mechanism where a good match between the physical and perceptual dimensions of the object is achieved.

## 1. Introduction

Size constancy in visual perception is a cognitive mechanism that allows us to perceive an object as having the same size even when seen at different distances [1,2] or when two objects at the same distance are seen from different perspectives, resulting in differential foreshortening. Indeed, we can maintain a veridical perception of the object as having the same physical properties, even though the retinal input may be different in each case. For instance, we perceive a dog as remaining the same size when it is standing nearby us and when it walks to a more distant location. Another example is when two objects are projected to different regions of the retina (i.e., fovea vs. periphery) with different levels of photoreceptor density and cortical magnification. It has been shown that an object appears smaller in the periphery than in the centre of the visual field [3]. It is suggested that this is due to the structural properties of the visual field [4] and, to some extent, a lack of spatial attention [5]. This issue of visual size constancy across different regions of the retina (fovea vs. periphery) has a very direct analogy with the issue of tactile size constancy across different regions of the skin with different mechanoreceptor densities, RF sizes, and cortical magnification [6,7,8].

Therefore, size constancy provides us with a stable perception of the world, although the retinal image is constantly changing, given that we and the objects around us move in the environment [9]. There is evidence that these cognitive mechanisms may be present from birth [10]. Similar to the tactile domain, due to the different sizes and shapes of tactile receptive fields (RFs) across body parts, compensatory mechanisms of tactile size constancy are necessary to allow a stable perception of touch. However, such mechanisms are sometimes incomplete, producing distorted representations of touch. Here, we provide empirical evidence suggesting that tactile constancy is determined by the nature of the stimulus.

There are several lines of evidence in the literature showing that visual size constancy is achieved not only by using information that derives from the retinal inputs but also from other extra-retinal signals that come from the eyes as well as other sensory organs [2]. For example, the eyes converge when a person directs their gaze on an object close to them and diverge when the gaze is directed to a more distant object. This process provides rich information to the brain about three-dimensional depth. Moreover, other sensory modalities such as audition [11] and proprioception [12] provide useful information about distance. For instance, when we hear a sound that is linked with a visual event (e.g., lightning), we can figure out the distance of the visual event by estimating the temporal gap between the two stimuli (vision and audition).

Critically, it has been recently shown that the primary visual cortex (V1) is an important area in which these different signals are integrated, well beyond the mere processing of retinal information [2], playing a key role in visual perception and perceptual constancy. Neuroimaging evidence in humans suggests that V1 activity better reflects the final perceived size of an object rather than the size of the retinal projection [13,14]. Research in how size constancy occurs in other sensory modalities, such as touch, has not been as extensive as for vision. However, recent evidence suggests that the primary somatosensory cortex (S1) can be considered a critical brain area that mediates tactile distance perception [15]. Like in visual studies of V1, activity in S1 closely mirrored the participant’s perception of stimulus size.

A classical illusion in tactile size perception is the one described by Weber in his pioneering studies [7], in which he found that the distance between two points touching the skin felt larger when applied to highly sensitive regions than to less sensitive regions. This phenomenon is known as Weber’s illusion. In the classical version of this study, Weber showed the presence of this illusion by comparing touches between different parts of the body. He found that there was a direct relationship between tactile size perception and tactile spatial acuity, a result that has been subsequently replicated several times by other studies [16,17,18,19].

Later research extended this notion by showing that analogous perceptual illusions are also present when the same region of the skin is stimulated using stimuli with different orientations [20,21,22,23,24,25]. In this respect, Longo and Haggard [22] found that the perceived distance between two touches on the width of the hand dorsum was about 40% bigger than the same distance along the length of the hand. This bias mirrors the anisotropy in tactile acuity on the limbs [7,26] and the shape of tactile receptive fields (RFs) [27,28]. Interestingly, it has recently been shown that such anisotropy is also present in the temporal domain, where temporal intervals between touches across the hand were perceived as longer than those along the hand [29]. Consistent with spatial anisotropy, temporal anisotropy did not appear on the palmar side of the hand. Moreover, changes in the perceived size of body parts produce corresponding changes in tactile distance perception [19,30,31], suggesting the need for a reference to a body model that includes the proportion of body parts [32].

Importantly, however, the magnitude of this illusion is significantly smaller than would be expected on the bases of sensitivity, cortical magnification, or RF geometry alone [22]. The origin of this anisotropy is suggested to derive from the RFs’ natural oval shape on certain body regions, such as the dorsum of the hand, and a process of tactile size constancy that compensates for the distortions incompletely [33]. In most previous studies investigating tactile anisotropy, this phenomenon was tested by presenting two distinct touches on the skin surface and asking people to make judgments about how far apart they felt. However, in everyday life, it is much more common for our skin surface to get into contact with objects rather than single touches. Therefore, it may be that additional information or cues in the tactile signal (i.e., a more substantial touch rather than just single points touching the skin) are necessary in order to accomplish complete constancy compensation, possibly through a lateral inhibition mechanism.

An exception is a study by Anema and colleagues [16] that used a modified version of Weber’s illusion in which a solid object was placed on the participants’ forearm and hand. They found a larger estimation of the size of an object on the palm of the hand than on the forearm, consistent with Weber’s illusion. Moreover, they also tested participants for grasping responses, which showed an opposite pattern, with a larger hand opening for objects when they were on the forearm [16]. They suggested that the greater aperture when grasping objects on the forearm than on the hand was due to greater uncertainty about object dimension because of the reduced receptor density in the arm. This result could be derived from the presence of a significant motor component in the task, namely, the fact that responses were given using the fingers’ aperture. Therefore, it remains unexplored whether the perceived size of an object can elicit the same amount of anisotropy on the dorsum of the hand that has been shown to be present when the distance between two touches has to be estimated in a pure perceptual context. Moreover, Anema and colleagues [16] asked participants to estimate the objects’ size in absolute value, whereas, in the present study, we will ask participants to provide a relative size estimation of the objects while they are presented on the same skin region at different orientations (i.e., across vs. along). This procedure allows us to directly quantify the amount of compensation necessary to correct the irregular shape of the tactile RFs.

To address this issue, the current study investigates whether the large and highly consistent distortions that emerge when participants have to estimate the distance between two touches on the skin also occur when the size of an object touching the skin surface has to be estimated. We measured the anisotropy of tactile size perception on the back of the hand [22]. In the distance condition, two pairs of touches defining different tactile distances were applied sequentially to the hand, one pair oriented proximodistally (along the hand) and the other oriented mediolaterally (across the hand). Participants made two-alternative forced-choice (2AFC) judgments of which distance felt larger. The method of constant stimuli was used to estimate biases in the perception of size as a function of orientation. Similarly, in the object condition, two pairs of objects defining different tactile sizes were applied sequentially to the hand, as above; in this case, the participants made 2AFC judgments of which size felt bigger. If the same extent of tactile size constancy applies to both the distance and size estimation of tactile stimuli, we should have the same level of anisotropy for both distance and size estimation. Instead, if a different extent of tactile size constancy mediates tactile distance and size estimation, the anisotropy should vary depending on the type of stimulation—i.e., points or objects.

## 2. Experiment 1

In the first experiment, we tested whether the anisotropy typically found in several studies investigating tactile size perception along versus across the hand dorsum using posts [20,21,22,25,34,35] is also present when stimuli are objects that stimulate the entire surface of the skin between two points. In all the experiments, participants with R-square values less than 0.5 were excluded from the analysis, as these indicate a poor fit of the data. This is the same criterion we have used in several other studies using this paradigm [35,36,37,38].

### 2.1. Material and Methods

Participants. Twenty participants (mean ± SD = 28.4 ± 9.7 years; 15 females) took part in Experiment 1. Participants reported normal or corrected-to-normal vision and normal touch. Participants were all right-handed except for one, as assessed by the Edinburgh Handedness Inventory [39] (M = 89, range −17–100). From previous studies conducted by Longo and colleagues using posts stimulation, investigating anisotropies in tactile distance perception on the hairy skin of the hand, a weighted average Cohen’s *d* was calculated of 1.51. We conducted a power analysis using G*Power [40] with this effect size, an alpha value of 0.05, and a power of 0.95, which indicated that 10 participants would be required for enough power. Therefore, our study is well powered to detect anisotropy for both posts and objects of comparable magnitude.

**Figure 1:**
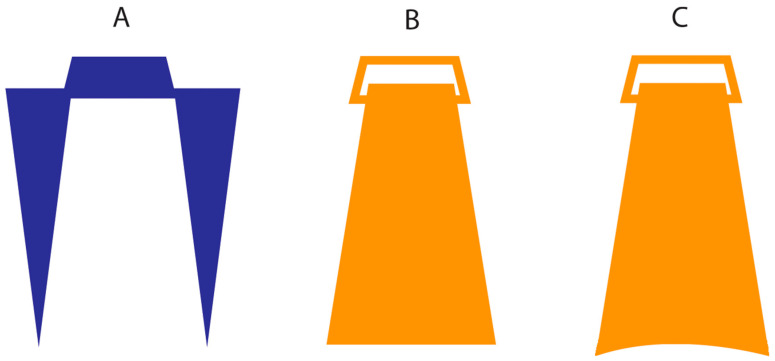
Schematic representation of the stimuli used in the three experiments: (**A**) posts, (**B**) flat and (**C**) curved objects’ surface. Note that the stimuli in panel (**A**) were used in all experiments. Stimuli in panel (**B**) were used for both the across and along stimulations in Experiment 1. In Experiments 2 and 3, stimuli in panel (**B**) were used for the along stimulation and stimuli in panel (**C**) for the across stimulation.

Stimuli. In half of the trials, stimuli were pairs of pointed wooden posts (diameter 1.5 mm) mounted in foamboard and separated by 2, 3, or 4 cm (Figure 1A), similar to those used in previous studies [22]. The tip of each rod tapered to a point but was not sharp. In the other half of the trials, stimuli were pairs of plastic objects of a shape that resembles that of an Isosceles triangle, to which a clip was attached to allow the researcher to hold it effectively with a good grip (Figure 1B). The lower surface of the object that touches the skin was slightly rough, with a length of 2, 3, or 4 cm and a width of 0.8 cm.

Procedure. The design and procedure were similar to many other studies [21,22,25,41]. On each trial, participants were touched twice on the dorsum of their left hand, once with the stimuli (i.e., posts or object, depending on the experimental block) oriented along the proximodistal axis of the hand (along stimulus) and once oriented along the mediolateral axis (across stimulus). Touch was applied approximately in the centre of the dorsum. Participants were instructed to make two-alternative forced-choice (2AFC) judgments of whether the two points or objects felt farther apart for the first or the second stimulus and responded verbally. There was no time constraint. In different blocks of trials, participants were touched using the posts or objects. We used five pairs of stimuli according to the size of the along and across stimuli (across/along): 2/4, 2/3, 3/3, 3/2, and 4/2 cm. Each pair was applied 12 times for a total of 120 trials per stimulation condition (i.e., posts or objects). The order of along and across stimuli was counterbalanced within each stimulus pair, the order of trials was randomised, and the order of experimental blocks (i.e., posts or objects) was counterbalanced across participants. The researcher administered stimuli manually with moderate pressure. The duration of each touch was approximately one second, with an inter-stimulus interval of approximately one second. Participants were blindfolded throughout the procedure and were not allowed to see the stimuli before testing commenced.

Analysis. The proportion of trials in which the stimuli delivered in the ‘across’ condition were judged as larger was analysed as a function of the ratio of the length of the across and along stimuli. To produce a symmetrical distribution of about the point-of-actual-equality (i.e., ratio equals 1), we used a logarithmic plot. Cumulative Gaussian functions were fit to each participant’s data with least-squares regression using the Palamedes toolbox for MATLAB [42]. The points at which the psychometric function crossed 50% were defined as the points of subjective equality (PSEs). The slope of the psychometric function was quantified as the inverse of the standard deviation of the best-fitting Gaussian. The data associated with this research are freely available on the Open Science Framework (OSF; osf.io/bqhx8 accessed on 28 February 2022).

### 2.2. Results and Discussion

R-squared values for the psychometric functions of individual participants ranged from 0.861 to 0.994 (M ± SE = 0.953 ± 0.01) with the posts and from 0.862 to 1 (M ± SE = 0.942 ± 0.01) with the objects, indicating comparable goodness of fit to the data for both types of stimulation condition (*t*(19) = 0.63, *p* = 0.53, *dz* = 0.14). Our main experimental question concerned the PSEs. If there is no distortion in tactile distance perception, PSEs should, on average, equal 1, indicating that stimulus orientation does not bias perceived distance. If there are distortions in tactile distance perception as for stimuli perceived far apart on the mediolateral hand axis, stimuli across the hand would need to be larger than those along the hand for the two to feel equivalent, and PSEs greater than 1 would be expected. Conversely, if the stimuli are perceived far apart in the proximodistal hand axis than they really are, stimuli along the hand would need to be larger than those across the hand for the two to feel equivalent, and PSEs less than 1 would be expected. Specifically, we were interested in testing whether these hand distortions may change as a function of the type of stimulus adopted (posts vs. objects).

**Figure 2:**
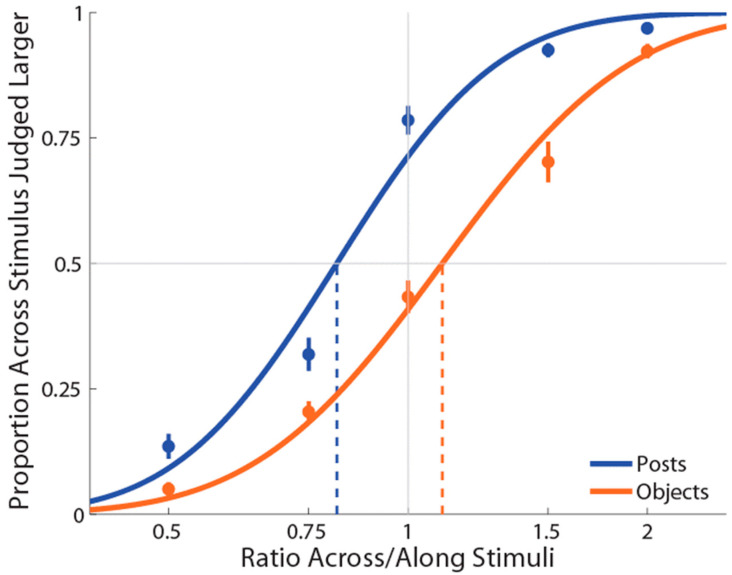
Experiment 1 results in which curves (blue for posts stimulations and orange for objects stimulations) are the cumulative Gaussian functions fit with least-squares regression. Vertical lines represent points of subjective equality (i.e., where the curve crosses 50%). Error bars represent the standard error of the mean (±SEM).

As shown in Figure 2, the mean PSE for the posts (M ± SE = 0.820 ± 0.02) stimulation condition was significantly less than 1 (*t*(19) = −7.09, *p* = 0.0001, *d* = 1.59), replicating the previously reported bias to judge distances as larger when oriented with the mediolateral than with the proximodistal axis [22]. By contrast, the mean PSE for the object (M ± SE = 1.11 ± 0.03) stimulation condition was significantly more than 1 (*t*(19) = 3.48, *p* = 0.003, *d* = 0.78), indicating an anisotropy in the opposite direction (i.e., for lengths aligned with the proximal–distal axis to be judged as larger than those aligned with the mediolateral axis). Moreover, we found that these distorted representations when posts or objects were used were significantly different from each other (*t*(19) = −8.51, *p* = 0.0001, *dz* = 1.90). There was no correlation between anisotropy in the two conditions, *r*(19) = 0.22, *p* = 0.34. Finally, the mean slope for posts (M ± SE ß = 6.3 ± 0.42) was significantly greater compared to the objects (M±SE ß = 5.4 ± 0.33) stimulation condition (*t*(19) = 2.51, *p* = 0.02, *dz* = 0.56), indicating that the task was more difficult with the objects than the posts.

These results, when using the posts stimulation, replicate previous reports showing similar anisotropy on the hand dorsum [21,22,23,24,25,34], with the presence of a clear bias for tactile distances to be perceived as larger when oriented mediolaterally, across the dorsum of the hand, than proximodistally, along the hand. However, when stimulated with an object, the typical effect not only vanished but also reversed in direction.

Our hypothesis was that using the objects may have caused a reduction of the anisotropy effect; however, we did not predict a reversal of the pattern. A possible explanation for the results we had when using the objects could be, at least to some extent, to backtrack to an artefact of the stimulation. The shape of the dorsum of our hand is not flat but slightly convex, particularly in the mediolateral axis rather than in the proximodistal axis. Therefore, the skin surface in these two directions has slightly different shapes. The fact that we used flat objects could have resulted in participants perceiving the across-presented objects (mediolateral axis) as smaller than they were just because their flat surface was not able to get fully into contact with the skin. By contrast, in the proximodistal dimension, the dorsum of the hand is almost flat; therefore, stimuli were properly applied. This issue is addressed in Experiment 2.

## 3. Experiment 2

As mentioned above, it is possible that the results of Experiment 1, when objects were used as stimulators, may in part derive from an artefact of the stimulation per se. To control for this possibility, in Experiment 2, we used different objects to stimulate the proximodistal (i.e., along) and mediolateral (i.e., across) hands’ axes. The former was the same we used in Experiment 1 (i.e., flat surface), whereas additional objects slightly bent were used to stimulate the dorsum in the mediolateral axis (Figure 1C). This has the effect of making the across and along objects stimulations equally effective in terms of the amount of surface of the skin touched. If the result of Experiment 1 when using objects is due, at least to some extent, to an artefact of the stimulation, we expect a reduction in the PSE value in the objects condition.

### 3.1. Material and Methods

Participants. Twenty participants (mean ± SD = 29.4 ± 10.4 years; 13 females) took part in Experiment 2. Nine participants were the same participants who had taken part in Experiment 1. Participants reported normal or corrected-to-normal vision and normal touch. Participants were all right-handed, as assessed by the Edinburgh Handedness Inventory [39] (M = 93, range 71–100).

Stimuli. These were the same as in Experiment 1 except for the fact that the objects stimuli used to stimulate in the mediolateral dimension were slightly bent (34 degrees) to better cover the whole skin surface.

Procedure. Procedures were identical to Experiment 1 except that the researcher used different stimuli, namely, flat or bent, when applying them to the across and along dimensions using objects, respectively.

### 3.2. Results and Discussion

One participant showed an extremely low R-squared value in the objects condition (0.178) and was excluded from subsequent analyses. For the remaining 19 participants, the average R-squared value for the posts condition was 0.954 (range = 0.837–1) and for the objects condition was 0.917 (range = 0.738–1), indicating a good fit to the data that was slightly better for the posts stimulation condition than the objects stimulation condition (*t*(18) = 2.49, *p* = 0.02, *d_z_* = 0.57).

**Figure 3:**
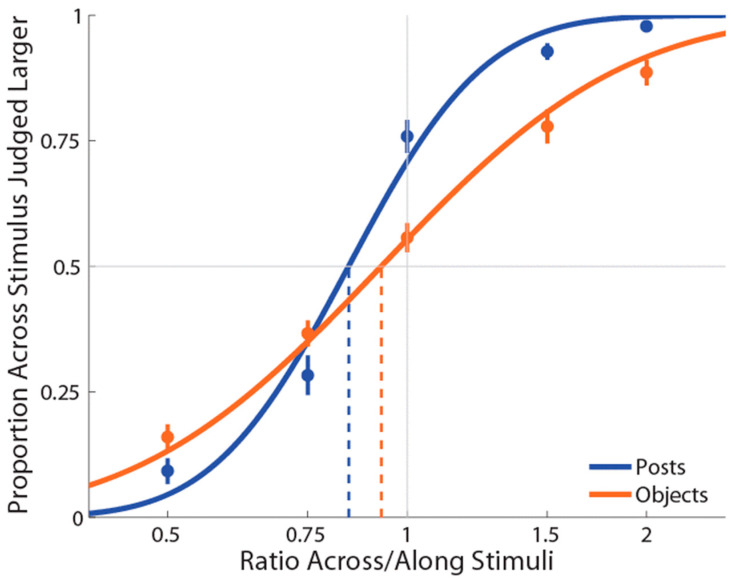
Experiment 2 results in which the curves (blue for posts stimulation and orange for objects stimulation) are the cumulative Gaussian functions fit with least-squares regression. Vertical lines represent points of subjective equality (i.e., where the curve crosses 50%). Error bars represent the standard error of the mean (±SEM).

As in Experiment 1, PSEs for stimuli presented using posts were again significantly less than 1 (M ± SE = 0.853 ± 0.03, *t*(18) = −4.87, *p* = 0.0001, *d* = 1.12; see Figure 3), indicating a large anisotropy. Further, the magnitude of bias was similar in the two experiments. Unlike Experiment 1, for the objects condition, we found that PSEs were lower than 1 (M ± SE = 0.937 ± 0.03, *t*(18) = −2.25, *p* = 0.037, *d* = 0.52) in the same direction as for the posts. Moreover, we found that these distorted representations for posts and objects were still significantly different (*t*(18) = −2.33, *p* = 0.03, *d_z_* = 0.54).

As for Experiment 1, the mean slope for the posts (M ± SE ß = 7.4 ± 0.58) stimulation condition was significantly greater compared to the objects (M ± SE ß = 4.2 ± 0.40) stimulation condition (*t*(18) = 6.15, *p* = 0.0001, *dz* = 1.41). There was no correlation between the two conditions, *r*(18) = 0.30, *p* = 0.22.

To compare the PSE values for the posts and objects conditions between Experiments 1 and 2, we performed a repeated measure analysis of variance (ANOVA) with type of stimulation (posts, objects) as a within-participant factor and Experiment as a between-participants factor. This analysis revealed the significant main effects of type of stimulation, *F*(1,36) = 51.76, *p* < 0.001, η_p_^2^ = 0.59, and of Experiment, *F*(1,36) = 4.20, *p* = 0.05, η_p_^2^ = 0.10, which were modulated by a significant interaction, *F*(1,36) = 14.16, *p* < 0.001, η_p_^2^ = 0.28. This derives from the fact that there was no differences in anisotropy for the posts between Experiment 1 and Experiment 2 (*t*(18) = −0.67, *p* = 0.50). However, for objects, the anisotropy was significantly different for Experiment 1 compared to Experiment 2 (*t*(18) = −3.92, *p* < 0.001).

These results replicate the anisotropy using the posts condition found in Experiment 1 and in many other studies. Moreover, it suggests that the reversed anisotropy seen in the objects condition of Experiment 1 may relate to a possible artefact created by the type of object used. Indeed, in Experiment 2, using bent objects for the mediolateral axis stimulation abolished the bias in representing the hand as narrower than it really is for this condition. Intriguingly, however, we found that the bias in representing the hand as wider than it really is was significantly lower in the objects condition compared to the posts condition. This indicates that the type of stimuli used, namely, posts or objects, may generate a different type of processing/compensation.

There is another substantial difference between the posts and objects conditions. Indeed, when we used posts of different sizes (i.e., 2, 3 and 4 cm), the same amount of skin surface was always stimulated. Instead, when we used the objects, increasing the length of the stimulus at the same time increased the amount of skin surface stimulated. This difference between the two types of stimulation may have, to some extent, contributed to the different biases in the two conditions. This issue is addressed in Experiment 3.

## 4. Experiment 3

In Experiment 2, we found that object stimulation produces anisotropy in the same direction as for the posts simulation; however, it was significantly reduced in its magnitude. A possibility is that this difference derives from the fact that in the objects condition, the surface area of the skin touched increases together with the length of the stimuli (i.e., 2, 3, 4 cm) since all stimuli have the same width. Therefore, the reduced anisotropy in the objects condition could be caused by the greater information provided to the skin by the object—i.e., more skin surface touched—in the objects condition compared to the posts condition. To test for this possibility, in Experiment 3, stimuli were the *posts*, as in the previous experiments, and two different types of objects. The first type of objects condition, named the *different-surface* condition, was as for Experiment 2, where the object’s length also increased as the dimension of the surface increased. In contrast, in the *same-surface* condition, when the object’s length was increased, the width was proportionally reduced, keeping the surface area of skin stimulated constant at 120 mm^2^. If the smaller anisotropy for the object than the post in Experiment 2 was due to the amount of surface touched, we should expect different anisotropies for the same- and different-surface stimulation conditions—i.e., different PSE values. By contrast, if the amount of surface stimulated is not the critical factor that causes this effect, we should not find any difference between the same and different conditions.

### 4.1. Material and Methods

Participants. Twenty participants (mean ± SD = 31.8 ± 13.8 years; 11 females) took part in Experiment 3. Four participants were the same participants who had taken part in Experiment 1 and Experiment 2. Participants reported normal or corrected-to-normal vision and normal touch. Participants were all right-handed, as assessed by the Edinburgh Handedness Inventory [39] (M = 94, range 48–100).

**Figure 4:**
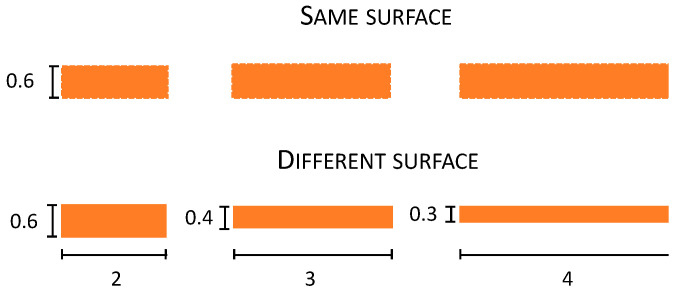
Depiction of the size of the different surfaces of the objects (same and different) used in Experiment 3. Numbers in the figures represent the dimension of the objects in centimetres (cm).

Stimuli. These were the same as in Experiment 2 except for the fact that there was an additional object condition (same surface) in which objects were weighted for their dimension (Figure 4). In particular, 2, 3, and 4 cm plastic objects were built to have the same surface area of 120 mm^2^, distributed for the three objects differently as a function of their dimension: 2 cm: 2 × 0.6, 3 cm: 3 × 0.4, and 4 cm: 4 × 0.3.

### 4.2. Results and Discussion

Two participants showed low R-squared values in the objects with the different-surface condition (0.451 and 0.461) and so were excluded from subsequent analyses. For the remaining 18 participants, the average R-squared value for the posts condition was 0.924 (range = 0.833–1), for the objects with the different-surface condition was 0.933 (range = 0.744–1), and for the objects with the same-surface condition was 0.937 (range = 0.783–1), indicating a good fit to the data.

**Figure 5:**
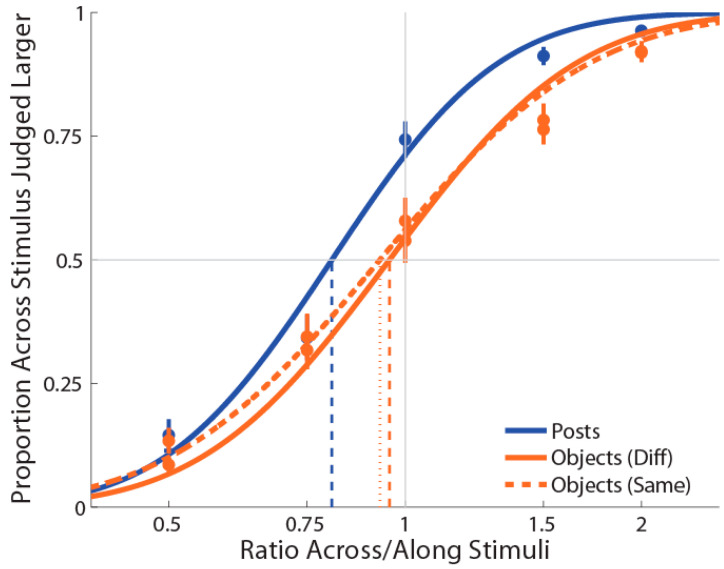
Experiment 3 results in which curves (blue for posts stimulations and orange for objects stimulations, dotted for the same surface) are the cumulative Gaussian functions fit with least-squares regression. Vertical lines represent points of subjective equality (i.e., where the curve crosses 50%). Error bars represent the standard error of the mean (±SEM).

As for previous experiments, PSEs for stimuli presented using posts were again significantly less than 1 (M ± SE = 0.816 ± 0.03, *t*(17) = −5.76, *p* = 0.0001, *d* = 1.36; see Figure 5), indicating a large anisotropy. Further, the magnitude of bias was similar to the previous experiments. For both the object conditions (i.e., same and different surfaces), we found that PSEs values were not significantly different from 1 (same surface: M ± SE = 0.950 ± 0.05, *t*(17) = −1.47, *p* = 0.16, *d* = 0.35; different surface: M ± SE = 0.974 ± 0.05, *t*(17) = −0.97, *p* = 0.35, *d* = 0.23). As for Experiment 1 and 2, we found that distance estimation for posts were still significantly different from both the objects with the same (*t*(17) = −3.07, *p* = 0.007, *d_z_* = 0.72) and different surfaces (*t*(17) = −3.54, *p* = 0.003, *d_z_* = 0.83). However, there was no significant difference between the same and different objects conditions (*t*(17) = 1.44, *p* = 0.17, *d_z_* = 0.34).

Moreover, the mean slope for posts (M ± SE ß = 6.0 ± 0.54) was significantly greater compared to same-surface objects stimulation conditions (M ± SE ß = 4.8 ± 0.40; *t*(17) = 2.12, *p* = 0.05, *d*_z_ = 0.50), but not for different-surface objects stimulation conditions (M ± SE ß = 5.3 ± 0.49; *t*(17) = 1.17, *p* = 0.25, *d*_z_ = 0.28). In addition, the mean slope for the different-surface objects condition was different compared to the same-surface objects condition, *t*(17) = 2.12, *p* = 0.05, *d*_z_ = 0.50. The mean slope between the two objects conditions were highly correlated, *r*(17) = 0.83, *p* = 0.0001. Moreover, the slope when posts were used were correlated with the same-surface objects condition, *r*(17) = 0.59, *p* = 0.01) but not with the different-surface objects condition, *r*(17) = 0.41, *p* = 0.09. The fact that the slopes of posts and different-surface objects were comparable may be an indication that the task was slightly easier in these conditions compared to the same-surface objects condition. We are not sure about the reason for such a difference, though it may have been caused by the fact that the amount of skin surface touched across different conditions was constant for the posts and different-surface objects conditions.

Overall, the results of Experiment 3 show once again the presence of anisotropy using the posts as stimuli. Moreover, we found that the bias in representing the hand as wider than it really is was significantly lower in the objects condition compared to the posts condition. Critically, this effect is not modulated by the amount of skin surface stimulated as we did not find any difference in terms of PSEs between the same- and different-surface objects conditions.

## 5. Statistical Comparison of the Three Experiments

To assess whether the PSE values for the posts and objects were consistent in the three experiments, we further performed a statistical comparison on our repeated measure designs. The aim of such analysis is to quantify across experiments the amount of anisotropy for the posts and objects. Data were analysed using R (version 3.3.3, with metaphor package, [43]).

**Figure 6:**
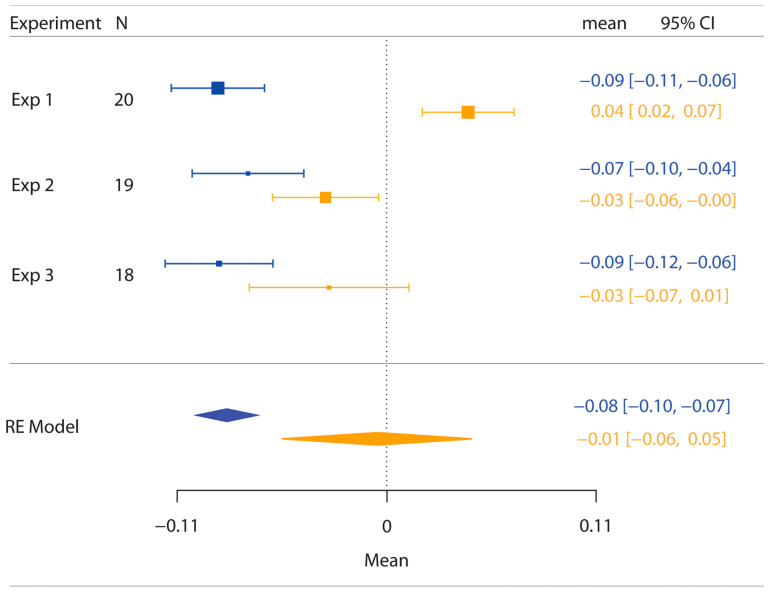
Plot of the results of the comparison across the three experiments of the PSE values for the posts (blue) and objects (orange) stimulation conditions. N represents the number of participants in each experiment. Error bars represent the 95% confidence interval (CI), which is reported on the left side of the graph for each condition across experiments in different colours. The random-effects model (RE Model) showed that the PSE for the posts condition is significantly lower than zero (*p* < 0.0001), whereas the PSE for the objects condition does not differ from zero (*p* = 0.8424). Note that differently from the graphs for the single experiments, here, the data are not plotted in log format.

As shown in Figure 6, a random-effects model (RE Model) comparing the PSE values for the posts across the three experiments resulted in the presence of significant anisotropy (M ± SE = −0.085 ± 0.008; z(−10.51), *p* < 0.0001), therefore confirming the bias in representing the hand as wider than it really is. By contrast, the same analysis of the PSE values for the objects condition did not reveal the presence of anisotropy (M ± SE = −0.005 ± 0.026; z(−0.199), *p* = 0.8424).

The results of this analysis further corroborate the notion that anisotropy is present only when the dorsum of the hand is stimulated with posts but not with objects.

## 6. General Discussion

In the present study, we found that two touches on the dorsum of the hand are perceived as larger when they are oriented mediolaterally (across the hand) than proximodistally (along the hand). Critically, however, when controlling for artefacts deriving from the stimulation, we found that this bias was significantly reduced (Experiment 2) or vanished (Experiment 3) when the stimuli were continuous objects. Moreover, the absence of this bias with continuous objects was not due to the curvature of the objects (Experiment 2) and was independent of the amount of skin surface stimulated (Experiment 3). The anisotropy we found when stimulating with the posts is in accordance with early [7] as well as more recent [22,26] reports that showed substantial anisotropy on the dorsum of the hand and in the shape of tactile RFs [27,28]. Therefore, our results for posts provide further evidence that such distortions may originate from primary somatosensory representations [15].

As discussed elsewhere by Longo and Haggard [22], such distortions are significantly reduced compared to what would be expected if they were simply reflections of the anisotropy of the RFs of individual neurons in SI. Therefore, it is likely that a process of tactile size constancy takes place to correct such distortions. In the case of objects, we propose that such a mechanism is simply more effective, given that the anisotropy was significantly reduced or absent. The somatosensory system thus appears capable of correcting substantially for anisotropic distortions but appears to do so less fully for distances than for continuous objects. In the next paragraphs, we will discuss a possible reason that may explain these effects and try to compare it with the size constancy effects and mechanisms that are currently proposed for visual perception.

The presence of anisotropy in tactile distance estimation when posts (Figure 1A) are used to stimulate the dorsum of the hand has been previously explained as a consequence of several factors, such as the sensitivity of the skin, cortical magnification, or RF geometry [33]. Moreover, given that such a factor would not be able to explain the magnitude of the anisotropy—i.e., such a phenomenon is reduced in magnitude compared to the difference in cortical magnification or tactile acuity—the authors proposed that a compensatory mechanism, though not complete, is taking place [22]. We propose that a possible reason that leads to a more substantial form of *tactile size constancy* when objects touch the skin rather than posts can be derived from a similar mechanism described for vision.

**Figure 7:**
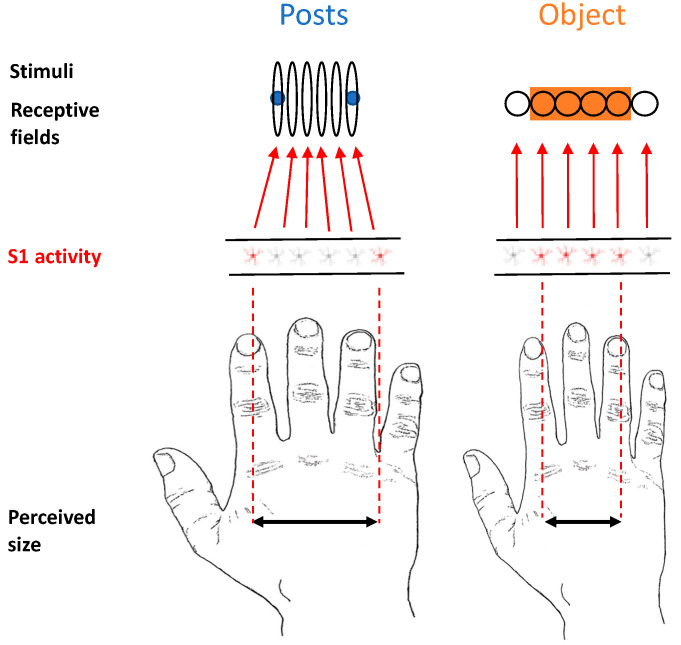
Depiction of the model that we propose of perceived size representation in the primary somatosensory cortex when objects are in contact with the skin surface.

We propose that when objects rather than posts contact the skin, “background” information deriving from a lateral inhibition mechanism triggers a process in which tactile RFs, which are actually distorted on the hand dorsum, shift location, reducing their natural oval shape and becoming more circular (Figure 7). In this respect, there is evidence in cats showing that injecting GABA antagonists in the primary somatosensory cortex produces asymmetric expansion of tactile RFs, making them even more elongated than they were at baseline [44]. This suggests that one consequence of lateral inhibition is to make tactile RFs more circular than they would otherwise be. This can be considered a natural potential mechanism of size constancy. We now describe the rationale of our reasoning, discussing two lines of evidence that point us in this direction.

First, we know that lateral inhibition mechanisms affect the way in which we perceive an object that touches our skin. This is well described by the tactile version of the well-known optical Müller–Lyer illusion [45]. In his book, von Békésy [45] suggests that this illusion in touch is created by a lateral inhibitory effect. In that study, a cardboard stimulus with a rounded edge was applied to the skin with a rocking motion. The participants perceived the edge of the form as more obtuse than the actual pressure. Von Békésy interprets this result as an effect of lateral inhibition, which shifts the perceived peak of the cardboard towards the aperture of the shape. Therefore, we know that lateral inhibition plays a key role in the context of perception of an object on the skin surface.

Second, as we have described above, the shape of the tactile RFs on the hand dorsum in several regions of the nervous system is not circular but oval [27,28,44], with the elongated part aligned with the proximodistal axis of the hand. It has been hypothesised that the tactile anisotropic effect for the posts can be due to this reason [33]. Tactile RFs have different sizes and shapes across the body; in addition, they have also different distributions of their excitatory and inhibitory subfields. DiCarlo, Johnson, and Hsiao [46], in a study on three alert monkeys, investigated the structure of tactile RFs in area 3b of the primary somatosensory cortex (S1) in terms of excitatory and inhibitory components of each RF. They studied neurons with RFs on the distal fingerpads with scanned random-dot stimuli. Overall, their results showed that most of the RFs (94% of the ones considered) contained a single central region of excitation. Interestingly, they also found that the RFs have one or more regions of inhibition located on one or more sides with respect to the excitatory centre. The shape area and strength of excitatory and inhibitory RF regions ranged widely. It is important to note that they reported that in 78% of the RFs, they found a continuous single region of inhibition positioned on one side of the excitation (52%), surrounding it partially (23%) or fully (3%). Interestingly, in the remaining 22% of the RFs, there were two regions of inhibition on opposite sides of the excitation [46]. Finally, these authors reported an overall distal displacement of the inhibitory subfields relative to the excitatory centre that coincided with the scanning direction.

Posts’ stimuli have a sharp tip; therefore, it is reasonable to expect that they were producing a homogenous deformation of the skin surface in all directions. By contrast, objects have a wider shape; therefore, they were probably producing a lateralised deformation in one or more directions of the skin surface. Such a difference in stimulation may have, in turn, produced a distribution of the inhibitory areas in the tactile RFs that was different across the two conditions. We hypothesise that a non-uniform distribution of the inhibition could somehow provide information regarding the shape of the object. We know that different responses can be elicited as a function of the type of surface touching the skin, at least on the fingertip [47,48,49]. Moreover, given the fact that tactile RFs on the hand dorsum have an oval shape, their inhibitory subfields may provide information about the actual shape of the RF itself. Namely, the elongated part in the proximodistal axis may have a greater inhibitory subfield in that direction compared to the not-elongated side. This information may allow the shape of the RF to be determined and, in turn, facilitate the computation of the actual skin surface stimulated. This may happen when an object is touching the skin and, therefore, continuously stimulating the RFs; however, this is not the case when the two posts touch the skin (i.e., uniform sharp touch). Such processing could have served as a tactile size compensatory mechanism that led to a greater correction for the distortions deriving from the tactile RFs’ shape in the case of the object compared to the posts. Therefore, lateral inhibition may provide information to overcome uniformity assumptions and produce a more effective compensatory process through a feedback signal sent to S1. This explanation would be compatible with a previously proposed hypothesis that suggests that distance and orientation on the skin are defined by the amount of skin intervening between two points [50].

Our tactile RF-shifting theoretical model (Figure 7) proposes that a lateral inhibition mechanism, when an object touches the skin, provides information through the distribution of the inhibitory subfields of the RF about the shape of the tactile RF itself. Such a process allows for an effective tactile size compensatory mechanism, where a good match between the physical and perceptual dimensions of the object is achieved. The fact that primary sensory areas play a critical role in size constancy has been shown in a recent study by Sperandio, Chouinard, and Goodale [14] in the visual domain. In this study, the authors showed that the retinal size of an afterimage activity in the primary visual cortex (V1) reflects the perceived image. The central role of S1 in complex processing such as tactile size constancy is in accordance with previous literature showing that this “sensory” area may not be critical for solving simple tactile tasks—i.e., tactile simple detection—in both monkeys [51] and humans [52]. By contrast, S1 seems to be critically involved in processing that was thought to be performed by higher-level cortical areas such as the bilateral integration of touch [53,54,55] as well as tactile working memory [56,57]. Moreover, in a recent fMRI study, we have shown that the tactile anisotropy on the hand dorsum seems to be mediated by the primary sensorimotor cortices [15]. Such intimate relations with the sensory and motor cortices may play a critical role in the control of finely tuned movements and complex motor skills. In this respect, Tamè and colleagues [53] found that activity in the somatosensory cortices (i.e., SI and SII) following repetitive (i.e., double) tactile stimulation causes finger-specific activation in the primary motor cortex, supporting the notion that spatial information is retained in SI and then transmitted to the motor cortex [58]. Such a relation between the sensory and motor systems is particularly relevant in the context of haptic tasks, in which we are required to actively explore an object.

In vision, it has been shown that responses in the primary visual cortex reflect perceptual outcomes rather than retinal inputs in both microelectrode recordings of individual neurons in monkeys [59] and fMRI responses in humans [14,60]. Recently, Ni and colleagues [61], in a very interesting experiment in monkeys, in which they measured visual RF responses, have shown that the firing of neurons in V1 reflects the perceived size of an object rather than its actual size. Using extracellular recordings, they showed that neurons in V1 can shift the position of their RFs when subjected to complex monocular depth cues. During the task, monkeys looked at rings over a Ponzo Illusory background (i.e., a corridor). In this context the illusion makes monkeys perceive the two rings as different in size (one bigger than the other), even though their actual physical size is the same. In particular, the rings positioned on the back of the corridor are perceived as bigger compared to the rings at the front. While monkeys looked at the ring at the back of the corridor, their vRFs shifted towards the centre of the rings. In contrast, when the rings appeared at the front of the corridor, their vRFs shifted outward. Ni and colleagues proposed that the size illusion derives from this shift of vRFs. Moreover, they suggested that such movement cannot be explained by the low-level features of the stimuli or by attentional factors. They suggested that visual RF movement is guided by higher-level stages of processing, which extract complex depth information from the picture background and then feedback this information to V1 to shift the position of the vRFs (see the model they proposed in Ni and colleagues [61]). A similar effect has also been found in a study conducted a year later in humans using fMRI-based population receptive fields (pRFs) in the visual cortex in combination with psychophysics [62]. Compatible with the work of Ni and colleagues [61], He and colleagues found that the far apparent rings in the scene caused the pRF positions of voxels in V1–V3 to shift toward the fovea, in line with participants’ percept of the Ponzo illusion. Moreover, they found that the pRF shift in V1 significantly correlated with the magnitude of the Ponzo Illusion [62].

Finally, results showed quite consistently across experiments that the task was more difficult for the objects than the posts. Such a result can be explained by the fact that in the objects condition, differently from the posts condition, the same regions of the skin are stimulated in the comparison between the across and along stimuli. Indeed, the centre of the dorsum was both touched by the across and along stimulation. Therefore, it may be that the partially same region of the skin stimulated twice may have created some confusion or a masking effect that generally reduced participants’ performance.

## Data Availability

The data associated with this research are freely available on the Open Science Frame-work (OSF; osf.io/bqhx8 accessed on 28 February 2022).
